# A zeolite-like aluminophosphate membrane with molecular-sieving property for water desalination†
†Electronic supplementary information (ESI) available: Experimental details and additional figures and tables. See DOI: 10.1039/c7sc04974a


**DOI:** 10.1039/c7sc04974a

**Published:** 2018-01-24

**Authors:** Yanju Wang, Xiaoqin Zou, Lei Sun, Huazhen Rong, Guangshan Zhu

**Affiliations:** a Faculty of Chemistry , Northeast Normal University , Changchun 130024 , P. R. China; b State Key Laboratory of Molecular Reaction Dynamics , Dalian Institute of Chemical Physics , Chinese Academy of Sciences , Dalian 116023 , P. R. China . Email: zouxq100@nenu.edu.cn

## Abstract

Membrane desalination has been proposed as a promising strategy to address the worldwide challenge of water scarcity. The development of membrane desalination is impeded by the limited availability of membrane materials, thus, selective membranes with high water fluxes are in high demand. Therefore, the synthesis of a zeolite-like aluminophosphate membrane for water desalination is reported herein. An AlPO_4_-18 membrane without visible cracks was prepared on stainless steel nets *via* a seeded approach. The specific adsorption and diffusion properties of the AlPO_4_-18 membrane toward water were simultaneously investigated by vapor adsorption measurements and molecular simulation. A large adsorption capacity indicates the excellent water affinity of the AlPO_4_-18 membrane, and water gathering around other water molecules suggests stronger water–water interactions than framework–water interactions. Meanwhile, water molecules transport rapidly through the pores, assisted by a built-in pathway and directed diffusion. The as-synthesized membranes are further evaluated for water desalination. The desalination tests of seawater containing both single and multi-component ions demonstrate that the AlPO_4_-18 membrane is very selective for water transport, evidenced by very high rejection degrees for cations (>99%). Moreover, the AlPO_4_-18 membrane exhibits unprecedented high water fluxes (2.14 kg m^–2^ h^–1^ in average). The superior performances of good selectivity, high water permeability and high stability ensure the potential application of supported aluminophosphate membranes in desalination.

## Abstract 2

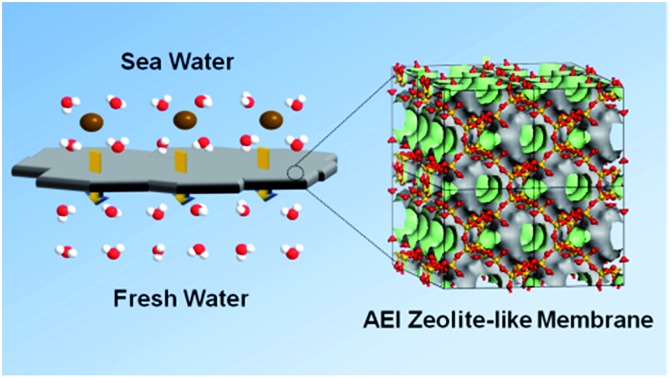
A fascinating membrane material composed of polycrystalline zeolite-like aluminophosphate with narrow pore and high water uptake is well developed, which exhibits superior desalination performance in terms of excellent ion rejection and record water flux.

## 1. Introduction

**Fig. 1:**
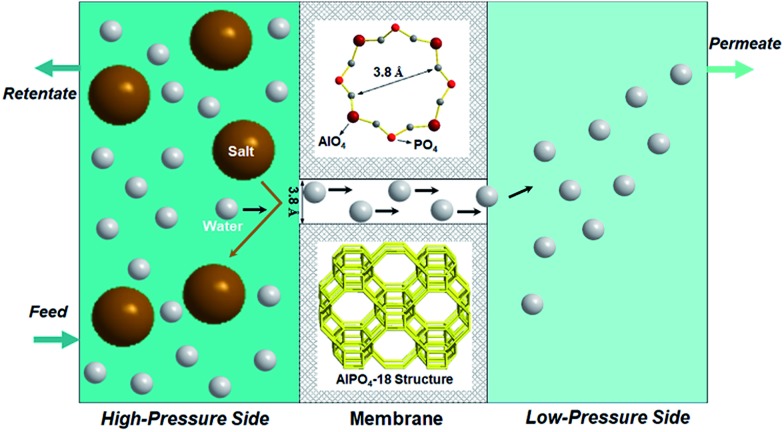
Schematic presentation of water desalination *via* membrane pervaporation process using the AlPO_4_-18 membrane.

Freshwater scarcity is becoming a great challenge for the sustainable development of our modern society because of intensive human activities and natural causes. Seawater desalination is one of the initiatives that address the problem of water shortage as a huge volume of seawater is available on our planet. Desalination works by selectively removing dissolved salts or minerals from seawater, brackish, and brine water to produce potable water for human consumption and domestic uses.^1^ Desalination technologies mainly encompass distillation-based thermal processes and membrane-based approaches. Membrane technology has attracted extensive interest owing to their inherent advantages such as energy efficiency and continuous operation.^2^ Membrane pervaporation is one of most promising desalination techniques due to its relatively low cost, simplicity and reliability, and module flexibility. As illustrated in Fig. 1, the working principle of the membrane pervaporation process is as follows:

The membrane acts as a selective barrier, separating the feed and permeate into two phases. When saline water is fed in, water passes through a semi-permeable membrane, driven by a pressure difference, while ions are simultaneously repelled in the retentate. Vaporized water is collected on the permeate side. The semi-permeable membrane is the core of the membrane pervaporation process, which determines water flux and ion rejection. However, limited membrane materials impede the development of membranes for desalination. Current membranes suffer undesirable tradeoffs between flux and ion rejection, namely, a highly permeable membrane has a low rejection and *vice versa*. In this regard, research in alternative membrane materials could enable more opportunities to balance flux and rejection in a better manner towards applications in desalination.

Among emerging membrane materials, zeolites have intrinsic advantages such as high permeability and good selectivity due to their well-defined pore structures and high porosities.^3^ Zeolite membranes are polycrystalline thin films commonly synthesized on rigid and porous substrates by *in situ* crystallization and seeded secondary growth methods.^4^ Aluminophosphates (AlPO_4_) as zeolite analogues are crystalline microporous materials with interesting properties and specific functions which impact our everyday lives, namely as catalysts and adsorbents.^5^ Similar to aluminosilicate zeolites, aluminophosphates are built from adjacent TO_4_ (T = Al, P) tetrahedra *via* corner-sharing to form ordered pores of molecular dimensions. Zeolitic membranes have found wide applications, particularly in gas or liquid separation. In the past two decades, intensive investigations have been carried out on zeolite membranes in terms of material discovery, synthesis renewal, and engineering technique advancement to enhance the separation performance.^6^,^7^ In parallel, continuous research efforts have been devoted to expand the applications of zeolite membranes beyond the realm of gas separation. The earliest simulation study in 2001 predicted that zeolite materials were perfect candidates for membrane desalination due to their uniform pore sizes and shapes.^8^ Subsequently, many membranes based on classical zeolites, including LTA, FAU, SOD and MFI types, have been experimentally prepared for water desalination.^9^–^30^ Unfortunately, limited applications were found for aluminophosphate membranes because of the poor membrane-formation ability. In recent studies, several aluminophosphate membranes have been attempted for gas separation.^31^–^33^ Motivated by the need for new materials for advanced applications, we herein aim to achieve the synthesis of aluminophosphate membranes and to extend their application to the new arena of seawater desalination.

In the present study, AlPO_4_-18 was selected as the target aluminophosphate material.^34^–^36^ AlPO_4_-18 has an AEI-type topology, which is constructed from equimolar AlO_4_ and PO_4_ units in strict alternation (Fig. 1). Consequently, the Al/P ratio in AlPO_4_-18 is close to unity and thus leads to a neutral framework. Additionally, AlPO_4_-18 possesses a three-dimensional 8-ring channel system, giving rise to pore openings of 3.8 Å and a cage diameter of ∼8.0 Å at the channel intersections. Small-size windows can maximize the molecular sieving effect to allow water molecules to pass through while excluding solvated salt ions based on their size discrepancies. In addition, the three-dimensional porous structure favours the formation of highly interconnected pathways for fast water transport (Fig. 1). The seeded method is employed for the synthesis of AlPO_4_-18 membranes owing to the advantages of decoupled nucleation and secondary growth during membrane crystallization. From a structural consideration, AlPO_4_-18 membranes are appealing for the separation of small molecules.

## 2. Results and discussion

### 2.1. Preparation and characterization of the AlPO_4_-18 membrane

**Fig. 2:**
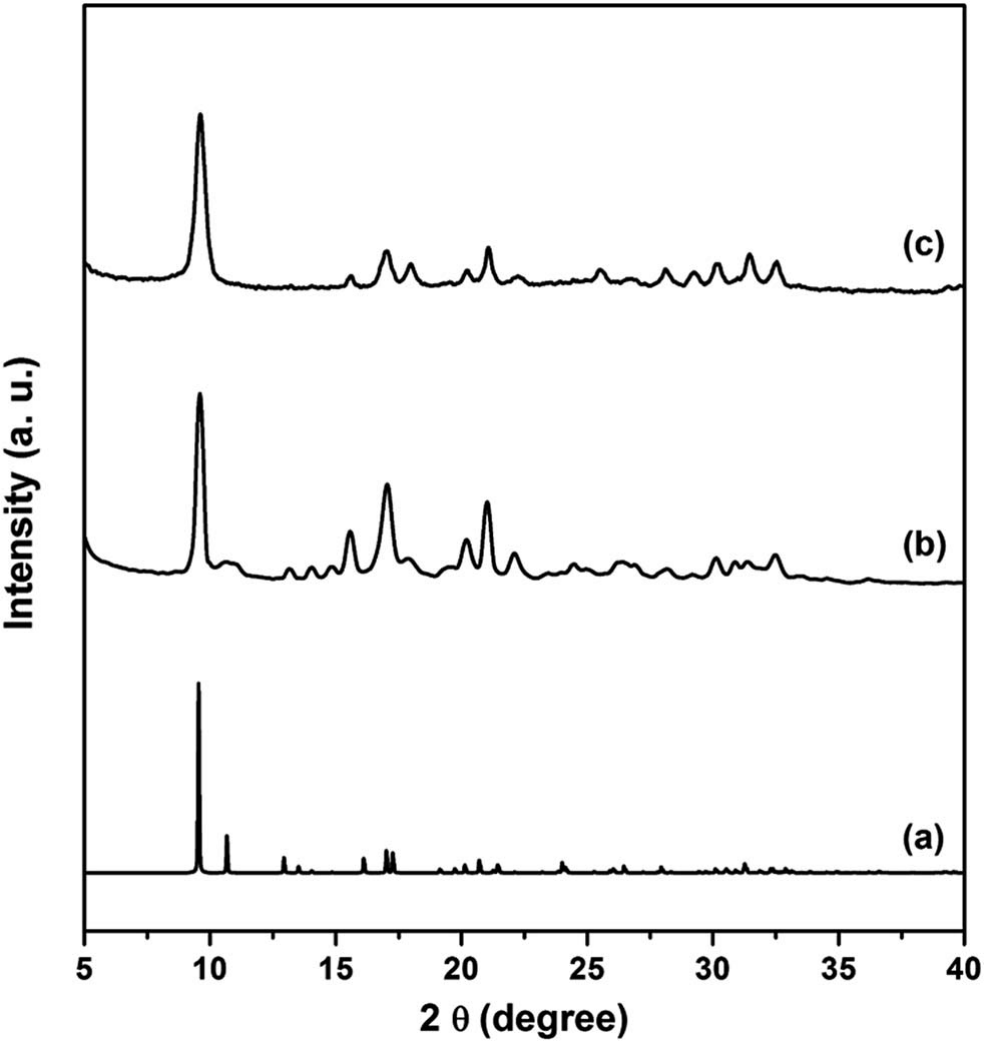
X-ray diffraction patterns of the (a) simulated AEI-type structure, (b) AlPO_4_-18 seed layer, and (c) as-prepared AlPO_4_-18 membrane.

The crystalline nature of the AlPO_4_-18 seeds and membrane layers were characterized by X-ray diffraction (XRD), and the patterns are collected in Fig. 2. As shown, all Bragg reflection peaks in both the seeds (Fig. 2b) and membrane (Fig. 2c) match well with the ones in the simulated pattern (Fig. 2a), indicating that the as-synthesized AlPO_4_-18 seeds and membrane have the same AEI-type crystalline structure.^34^ No extra peaks were detected in the diffractograms of neither the seeds nor the membrane layers, indicative of pure AlPO_4_-18 crystals without any trace of impurities.

**Fig. 3:**
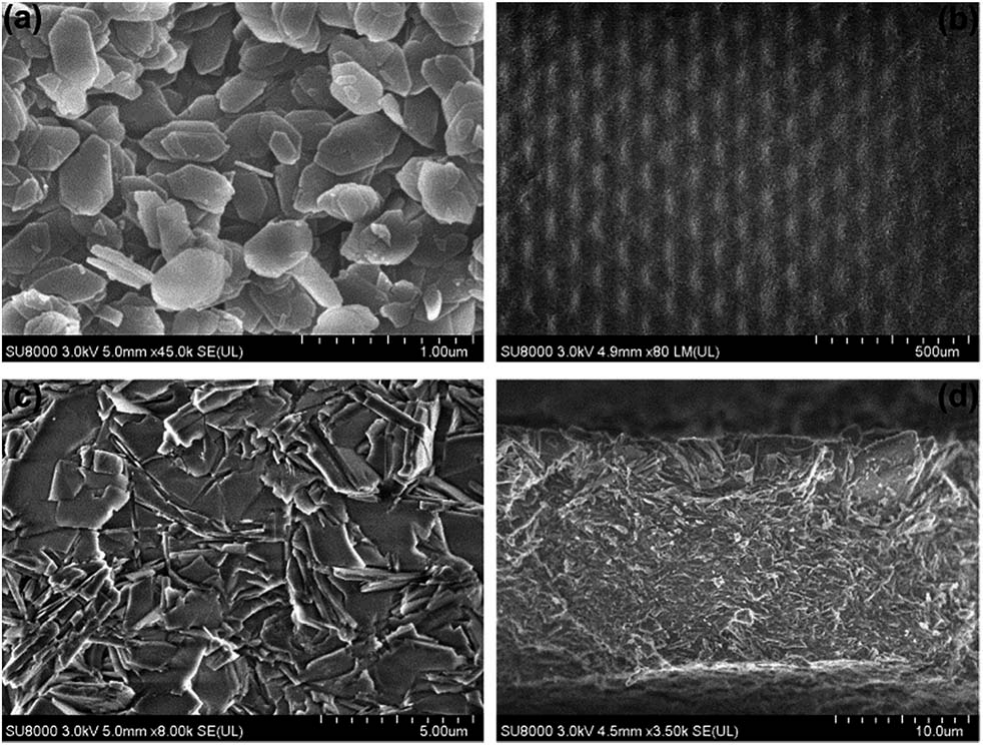
SEM images of (a) the AlPO_4_-18 seed layer deposited on the stainless steel net, (b and c) top views of the synthesized AlPO_4_-18 membrane with low and high magnifications, and (d) side view of the supported membrane.

The morphologies of the AlPO_4_-18 seed layer and membrane were inspected in detail by scanning electron microscopy (SEM), and the SEM pictures are shown in Fig. 3. As can be seen, the AlPO_4_-18 seeds display hexagonal plate-like shapes, and the sizes are estimated to be around 500 nm (Fig. 3a). Meanwhile, the net support is homogenously covered by a continuous layer of AlPO_4_-18 nanocrystals. The full coverage of the support by the seeds can promote uniform crystallization during the secondary-growth stage to facilitate the formation of a continuous membrane. The as-prepared AlPO_4_-18 membrane after hydrothermal treatment was also monitored by SEM. From the top view (Fig. 3b), a continuous AlPO_4_-18 membrane is obtained after an additional 24 h-treatment in the synthesis solution. The secondary crystallization of the AlPO_4_-18 seeds has two consequences: crystal enlargement and intergrowth.

As shown in a magnified SEM image in Fig. 3c, individual crystallites in the membrane are approximately 3.0 μm in length, which are significantly larger than the seeds (500 nm). Due to limited space on the support, the AlPO_4_-18 crystals undergo an intergrowth process during the secondary crystallization. This overgrowth causes healing of the grain boundaries between neighboring crystals. Both phenomena account for high continuity and good compactness, supported by the polycrystalline AlPO_4_-18 membrane without visible cracks (Fig. 3c). Fig. 3d shows the cross-sectional image of the membrane from which a thickness of approximately 15 μm is determined. With a close look, we can observe that plate-like AlPO_4_-18 crystals are inter-grown in a stack-by-stack mode.

More structural properties were further investigated by combined techniques, and the texture data is summarized in Table S1.† The Al/P ratio in the as-synthesized AlPO_4_-18 membrane is measured by ICP to be 1.02, confirming the equivalence of Al and P atoms in the framework. The surface area of the AlPO_4_-18 crystals collected from the membrane is calculated to be 754.0 m^2^ g^–1^. The pore size is estimated to be 8.23 Å, corresponding to the crystallographic value of the AEI-type cavity. A total and micropore volumes of 0.647 and 0.283 cm^3^ g^–1^ are also determined by nitrogen physical sorption.

### 2.2. Water adsorption property of AlPO_4_-18

**Fig. 4:**
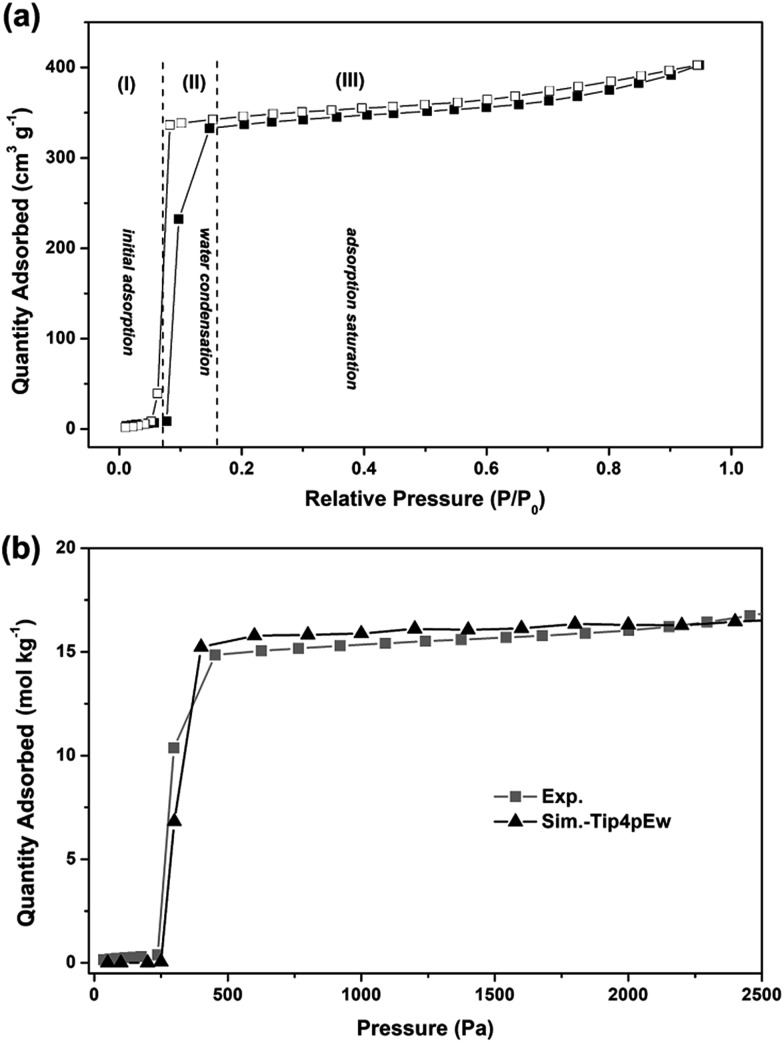
(a) Measured adsorption–desorption isotherms of water on AlPO_4_-18 collected from the membrane (filled and empty symbols represent adsorption and desorption branches, respectively; *P*_0_ refers to the saturated water vapor pressure at 298 K; stages I–III display initial adsorption, water condensation, and adsorption saturation; dashed lines present corresponding thresholds), and (b) comparison of experimental and simulated adsorptions for water molecules in AlPO_4_-18 at 298 K.

Fig. 4a shows the measured water sorption isotherm of AlPO_4_-18 at 298 K. The adsorption isotherm exhibits a three-stage uptake profile. In detail, a very small uptake of water is measured at low pressures (*P*/*P*_0_ ≤ 0.08), and the adsorption branch is convex with respect to the *P*/*P*_0_ axis (stage I). Both observations indicate that the attractive interaction between the water molecules and the pore walls is relatively weak, which can be interpreted by the small number of adsorption sites in the neutral AlPO_4_-18 framework. A rapid increase in the adsorption amount (stage II) is observed at the relative pressure range of 0.08 to 0.15, reflecting that interactions between water molecules govern the adsorption at medium pressures. The consequences of strong water–water interactions and weak water–pore interactions lead to the occurrence of water condensation in the confined spaces of the AlPO_4_-18 cavities.^37^ The limiting uptake at high *P*/*P*_0_ (stage III) leads to a plateau in the isotherm, which suggests complete pore filling. A small hysteresis loop is also measured between the adsorption and desorption branches, suggesting the development of metastable water fluids (*e.g.* water clusters) in the AlPO_4_-18 cavities. This phenomenon can be explained by the large size of the cavity (8.23 Å) and the narrow pore opening (3.8 Å) in the AEI-type framework. The hysteresis closure indicates the physical adsorption of water in the AlPO_4_-18 pores, which is beneficial for rapid water transport through the membrane *via* an adsorption–desorption process.

To microscopically elucidate the sorption behavior, molecular simulations of water adsorption on AlPO_4_-18 were performed.^38^–^42^ The simulated isotherm of water adsorption in AlPO_4_-18 at 298 K is shown in Fig. 4b.

**Fig. 5:**
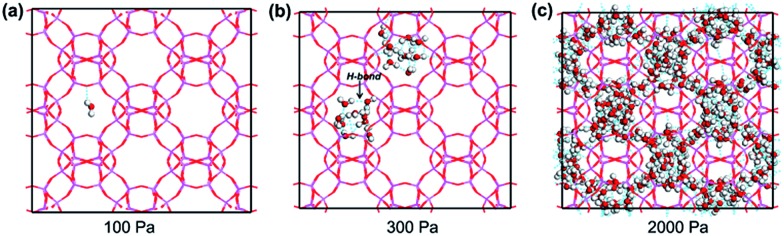
Snapshots of water adsorption in AlPO_4_-18 at 298 K and (a) at 100 Pa, (b) at 300 Pa, and (c) at 2000 Pa water (the dotted line represents intermolecular hydrogen bonds).

It can be seen that the simulation reproduces the experimental adsorption isotherm. At low pressures (below 250 Pa), water molecules are hardly adsorbed into the pores. For example, the average molecule loading excess at 100 Pa is only 0.008 molecules per unit cell (Fig. 5a). Clearly, the water–water interactions are stronger than the water–framework interactions. With increasing pressure, the isotherm shows a vertical jump in the water sorption around 300 Pa, at which the average molecule loading excess is 20.40 molecules per unit cell. This behavior is mainly due to the occurrence of condensation transition. The water molecules bind together through intermolecular hydrogen interactions between oxygen and hydrogen atoms as shown in Fig. 5b. With a further increase in pressure, the water uptake slowly increases. At 2000 Pa, the average molecule loading excess is 48.75 molecules per unit cell. Water molecules fill the large pores, and the vapor-like water gradually transforms into liquid-like water as shown in Fig. 5c. The simulation result reflects the cooperative effect of the water molecules, which has been described in previous works.^43^,^44^ When several water molecules adsorb in the pore, they behave as active sites where more water molecules assemble through hydrogen bonding, due to the stronger interactions between the water molecules. Based on the maximum equilibrium uptake of 16.5 mol kg^–1^, we determined the number of water molecules per cage to be 12 (assuming a homogeneous distribution of water molecules in AlPO_4_-18 cages, four cages per unit cell, *V*_cell_ = 3.19 nm^3^),^34^ which means that each AlPO_4_-18 cavity is occupied by a twelve-water cluster.^37^ The high occupancy allows frequent water transport from one cage to another in the membrane.

### 2.3. Desalination with AlPO_4_-18 membrane

**Fig. 6:**
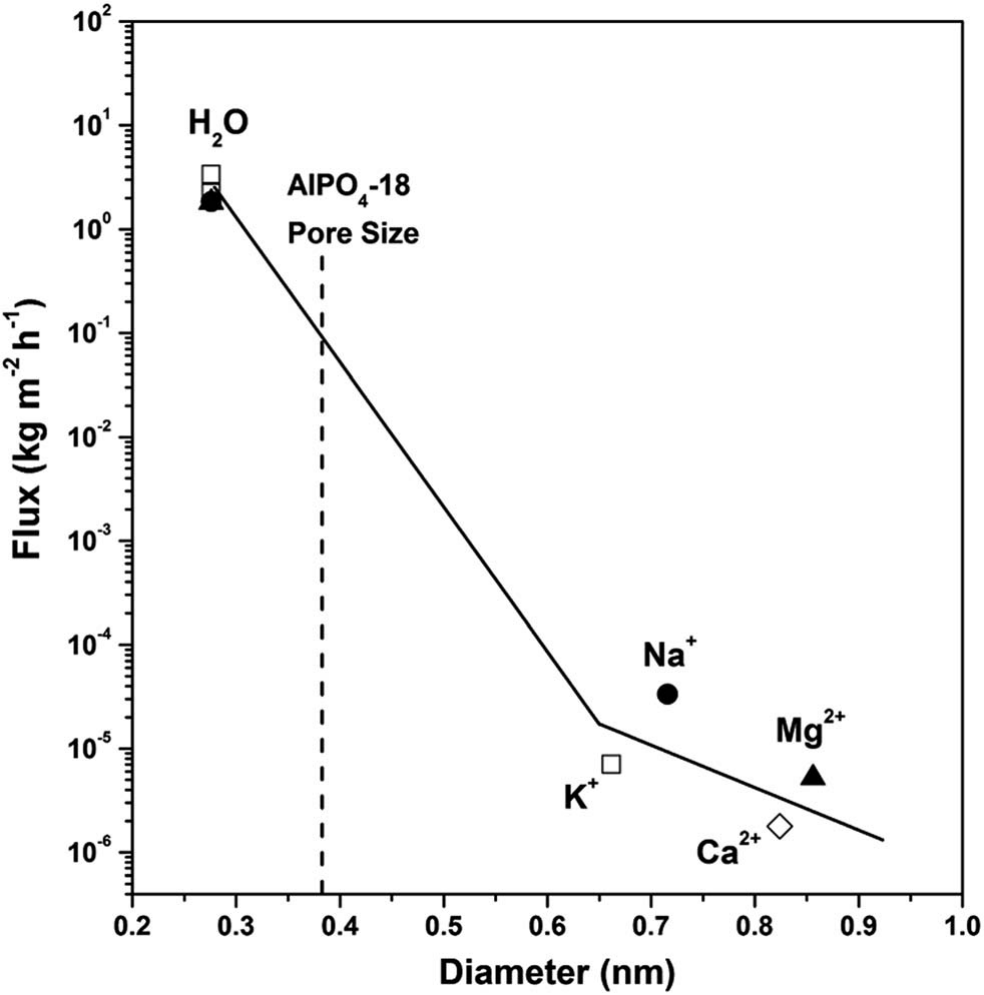
Flux of water and cations in single-component aqueous solutions (0.5 M for each salt solution, feed pressure of 1.0 atm) on the calcined AlPO_4_-18 membrane at 298 K as a function of species kinetic diameters.

Prior to desalination, the AlPO_4_-18 membrane continuity was checked by the permeation of H_2_ and N_2_ gas in standard mode. The H_2_/N_2_ selectivity of 7.6 is far beyond the Knudsen coefficient (3.7), proving that a minimum number of defects is present in the membrane. Furthermore, the potential for desalination of the membrane was exemplified by the transport of water and metal ions (Na^+^, K^+^, Mg^2+^ and Ca^2+^, which are the main components of seawater). Fig. 6 gives the fluxes of H_2_O, Na^+^, K^+^, Mg^2+^ and Ca^2+^ in single-component aqueous solutions through AlPO_4_-18 membranes as a function of the kinetic diameters of the water molecule and solvated cations. Water flux is almost 10^5^ orders of magnitude higher than those of the cations (Na^+^, K^+^, Mg^2+^ and Ca^2+^), and there is a clear cut off in the fluxes between H_2_O and K^+^. On the basis of cation concentration in the feed and permeate solutions, rejection degrees of 99.87%, 99.96%, 99.97% and ∼100.00% are calculated for Na^+^, K^+^, Mg^2+^ and Ca^2+^, respectively (Table 1). This experimental result verified our previous hypothesis that water and cation transport can be kinetically controlled, and the size-exclusion effect can be fulfilled by using ultramicroporous AlPO_4_-18 membranes. That is to say, only small water molecules (2.76 Å) can pass freely across the narrow windows of the AlPO_4_-18 membrane (pore size of 3.8 Å) to the permeate side, while big solvated cations are excluded in the feed side (7.16 Å, 6.62 Å, 8.56 Å and 8.24 Å, for Na^+^, K^+^, Mg^2+^ and Ca^2+^, respectively). The high rejection degrees (>99% in Table 1) also show that minimal non-size-selective mass transport exists in the AlPO_4_-18 membrane. Desalination tests of salts in a simulated seawater solution for the AlPO_4_-18 membranes were implemented as well. The water fluxes and rejection degrees are summarized in Table 1. As can be seen, the water flux in multi-component seawater is similar to that in the single-component solutions, which is a sound proof that water transport is not influenced by co-existing cations. The high rejection degrees for all cations (close to 100%) indicate an independent mass transport mechanism for individual components in simulated seawater, which can be correlated with the AlPO_4_-18 structure with small pore opening.

**Table 1:** Water flux and cation rejection degree in single- and multi-component aqueous salt water on the AlPO_4_-18 membrane at 298 K and with a pressure drop of ∼1.0 atm

Feed solution	Concentration (mol L^–1^)	Water flux (kg m^–2^ h^–1^)	Cation rejection (%)
NaCl	0.5	3.37	99.87
KCl	0.5	2.23	99.96
MgCl_2_	0.5	1.82	99.97
CaCl_2_	0.5	1.84	100.00
Seawater (3.5% salts)			
Na^+^	0.5	2.14	99.70
K^+^	0.01		99.95
Mg^2+^	0.05		100.00
Ca^2+^	0.01		99.99

**Fig. 7:**
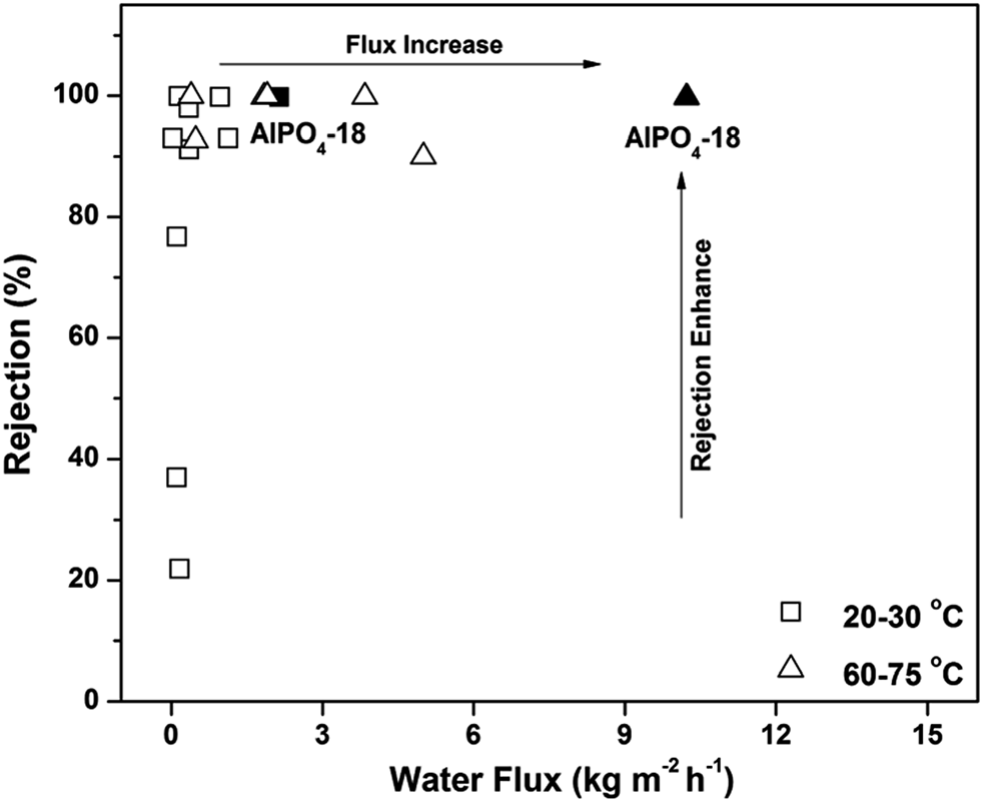
Rejection degree *versus* water flux for various polycrystalline zeolitic membranes used in water desalination (test conditions: 20–30 °C or 60–75 °C, pressure drops of 0.1–2.76 MPa, salt concentrations of 0.04–0.5 M). The open symbols refer to previous reports, and the filled symbol refers to this study.

**Fig. 8:**
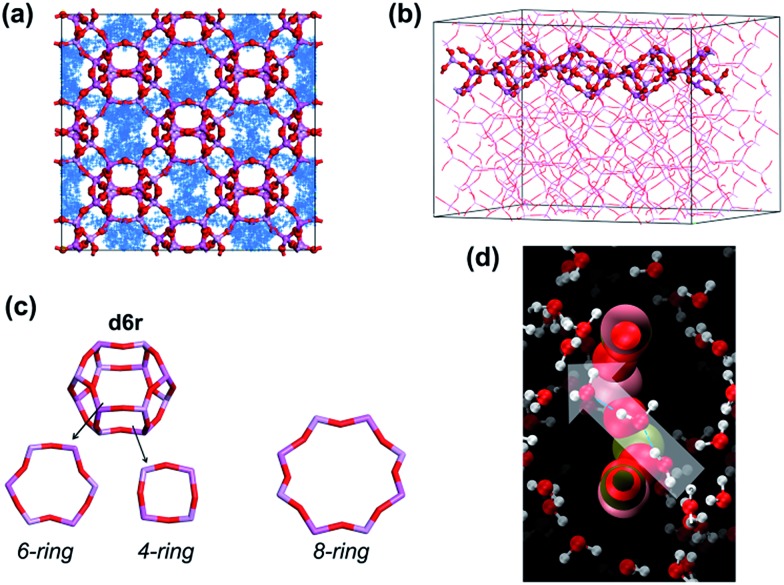
Dynamic trajectory of one water molecule in AlPO_4_-18: (a) view of the *x*–*y* plane, and (b) side view of *d6r* building unit in AlPO_4_-18; (c) individual *d6r* building units and ring structures (4-ring, 6-ring, and 8-ring); and (d) snapshot of water diffusion through a single AlPO_4_-18 pore. Points in (a) represent the dynamic trajectory; in (d), the dotted line represents water pathway; for guidance, the arrow shows diffusion direction from one side to another.

Notably, the water flux of the AlPO_4_-18 membrane herein is a new benchmark in polycrystalline zeolite membranes (Fig. 7),^19^–^30^ and its value (water flux of 2.14–3.37 kg m^–2^ h^–1^ bar^–1^ at rejection of >99.5%) also reaches the upper limit of currently available membranes for water desalination (2.5 L m^–2^ h^–1^ bar^–1^ at 100% rejection).^45^ The exceptionally high water flux can be rationalized using the descriptors of high solubility (*S*) and fast effective diffusivity of water (*D*_eff_). The high adsorption capacity improves the water solubility and thus leads to frequent water transport. The effective water diffusivity is controlled by its intrinsic diffusivity, diffusion length and transport direction. To prove this hypothesis, simulated studies were conducted.^46^,^47^ The dynamic trajectory of one water molecule in AlPO_4_-18 is shown in Fig. 8a. The points represent the MD trajectory. As can be seen, the water molecule can move freely among the large pores. It is also found that small pores (composite building units of *d6r*) in the framework are not filled (Fig. 8a). As seen from another direction (Fig. 8b), the *d6r* units consisting of 6-rings and 4-rings (Fig. 8c) are empty during the dynamic process. The smaller diameters of the 6-ring and the 4-ring prevent molecule transition. Therefore, the water molecules diffuse only through the bigger 8-rings in AlPO_4_-18, validating the molecular sieving effect of the membrane.

The self-diffusion coefficients (*D*_s_) for m1, m8 and m396 are 12.06 × 10^–9^, 0.33 × 10^–9^ and 0.08 × 10^–9^ m^2^ s^–1^, respectively. With increasing molecule loading, the *D*_s_ of water decreases. Upon analyzing the dynamic process, we find that only one water molecule passes through the 8-ring at a time, which supplements the single molecular transport mechanism. The molecular diffusion is limited by the narrowness of the pore opening (8-ring), especially when more water molecules are introduced into the AlPO_4_-18 pores. However, an efficient water transport pathway is prone to be built on extensive water molecules in cavities as well as at pore mouths, which can be vividly observed in Fig. 8d. This pathway building can shorten the diffusion length of water from one position to another and consequently increase the water diffusion rate. To support this conclusion, the energy barrier for water permeation through the AlPO_4_-18 membrane was determined from the temperature-dependent fluxes (Fig. S1†). The activation energy is measured to be ∼20 kJ mol^–1^, which lies within the hydrogen-bond energies of 14–21 kJ mol^–1^ for vaporized water.^48^ This value is even lower than that of highly hydrophilic FAU zeolites.^22^ Moreover, the direction of water movement can be controlled by applying an external pressure across the membrane in the permeation experiments.

From both the computational and experimental studies, it can be concluded that the large adsorption capacity contributes to the high water solubility, and the synergistic factors of relatively fast intrinsic diffusivity, short diffusion length and directed water transport participate in increasing the effective diffusivity. The increase in solubility and diffusivity is an outstanding improvement in the overall water transport rate over the AlPO_4_-18 membrane.

**Fig. 9:**
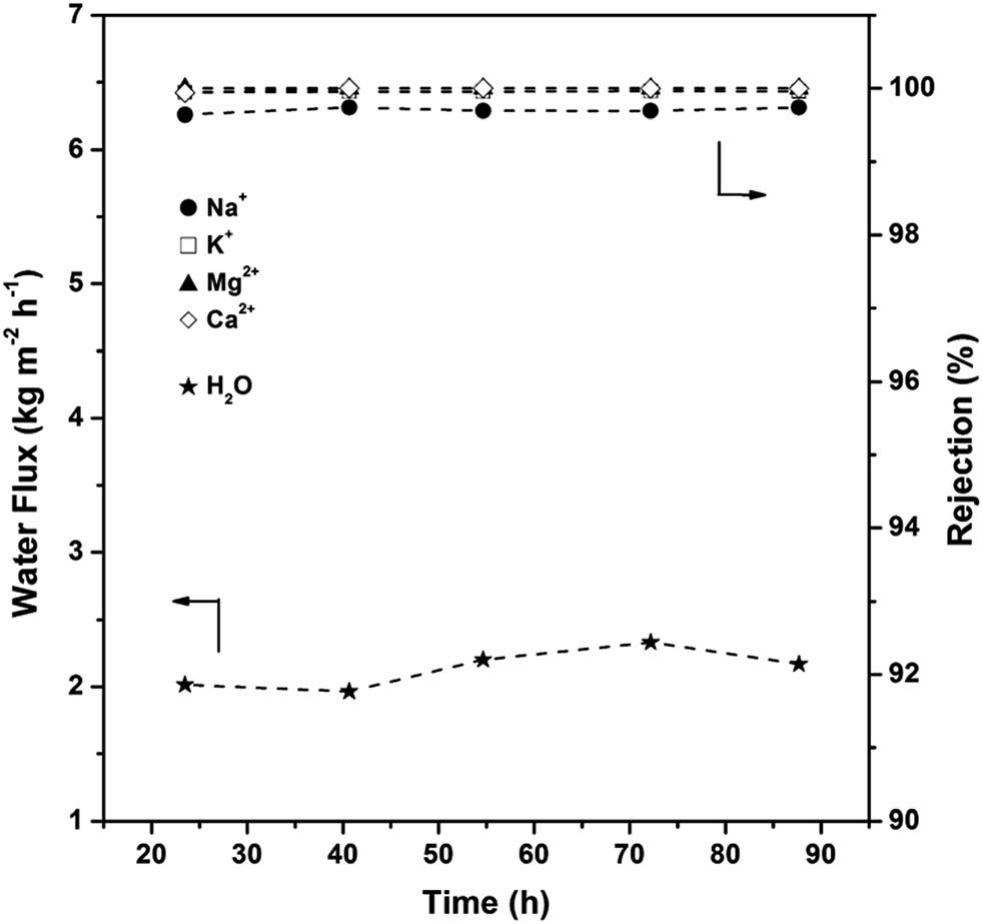
Water flux and cation rejection degree of the AlPO_4_-18 membrane in simulated seawater as a function of test time (test conditions: 298 K, feed pressure of 1.0 atm).

To address the robustness and reproducibility of the membrane, water permeations for different time periods were evaluated. As shown in Fig. 9, the AlPO_4_-18 membrane retains the water flux (2.14 ± 0.1 kg m^–2^ h^–1^) and the rejection degrees (99.7% for Na^+^, 99.9% for K^+^, 100% for Mg^2+^, 100% for Ca^2+^) over a 90 h period. The membrane can be used repeatedly over a long period of time, showing the high stability of the supported membrane, which is applicable in seawater desalination. It is worth mentioning that the cation rejection degree (Fig. S2†) reaches a steady state (99–100%) in a short time (<6 h), which sheds light on the perfectly inter-grown membrane without grain boundaries and the neutral framework. This data is consistent with negligible concentrations of cations remaining in the AlPO_4_-18 membrane after the desalination test (Fig. S3†).

## 3. Conclusions

In conclusion, high-quality AlPO_4_-18 zeolite-like membranes were successfully fabricated on stainless steel net supports. The continuity of the AlPO_4_-18 membrane was achieved by an intergrowth of AlPO_4_-18 crystals in the membrane layer during the secondary crystallization. The as-synthesized AlPO_4_-18 membranes were further tested for water desalination. The desalination results revealed preferential permeation of water molecules over solvated cations through the AlPO_4_-18 membranes, evidenced by high cation rejection degrees. The high selectivity of water transport arose from the molecular-sieving effect due to the small pore apertures of AlPO_4_-18. Furthermore, the as-prepared membranes have demonstrated unexpectedly high water fluxes. The particulars of water permeation were studied in detail by the combined techniques of pervaporation measurements and molecular simulations. On the basis of the experimental and theoretical results, a possible mechanism for membrane desalination was deduced: the high water capacity ensured frequent and facilitated mass transport across the membrane, making a major contribution to the flux. In parallel, the water-transport-pathway building, shortened diffusion length, and directed water diffusion contributed substantially to the enlarged effective diffusivity. The product of the increased sorption ability and enlarged diffusivity eventually enhanced the overall water flux through the AlPO_4_-18 membrane. In addition, the supported AlPO_4_-18 membranes displayed high robustness and reproducibility, undoubtedly giving this membrane material immense promise in water desalination.

## Conflicts of interest

There are no conflicts of interest to declare.

## Supplementary Material

Supplementary information
