# Supplementary material for: A zeolite-like aluminophosphate membrane with molecular-sieving property for water desalination
Source: Chem Sci. 2018 Jan 24;9(9):2533–9. doi: 10.1039/c7sc04974a (PMC5909670; doi:10.1039/c7sc04974a)
Supplement: Supplementary file 1 [file SC-009-C7SC04974A-s001.pdf]

Electronic Supplementary Material (ESI) for Chemical Science.
This journal is © The Royal Society of Chemistry 2018

Supporting Information

Zeolite-like aluminophosphate membrane with molecular-sieving property for water desalination

Yanju Wang,[a] Xiaoqin Zou,[a,*] Lei Sun,[b] Huazhen Rong[a] and Guangshan Zhu[a]

[a] Faculty of Chemistry, Northeast Normal University, Changchun 130024, P. R. China.

[b] State Key Laboratory of Molecular Reaction Dynamics, Dalian Institute of Chemical Physics, Chinese Academy of Sciences, Dalian 116023, P. R. China.

*E-mail: zouxq100@nenu.edu.cn

1. Experimental

1.1. Materials

Chemical reagents were of analytical grade and used as received without any further purification: aluminium isopropoxide ($Al(i\text{-}C_3H_7O)_3$, Tianjin Guangfu Chemical Reagent Co., Ltd.), tetraethylammonium hydroxide (TEAOH, 35 wt% aqueous solution, Alfa Aesar), phosphoric acid (H_3PO_4, 85 wt%, Beijing Chemical Reagent Company), salts of NaCl (99.5%, Beijing Chemical Reagent Company), KCl (99.5%, Tianjin FuChen Chemical Reagent Factory), $MgCl_2$ (98%, Tianjin DaMao Chemical Reagent Factory) and $CaCl_2$ (96%, Beijing Chemical Reagent Company). Na^+, K^+, Mg^{2+} and Ca^{2+} (0.5 mol L^{-1}, 0.5M) single and multi-component solutions (0.57M, ~3.5 wt%) were prepared by dissolving the corresponding salts in deionized water.

The substrate of porous stainless-steel-net (300 mesh) was purchased from State No. 540 Company, Xinxiang, China.[18] Prior to use, the net was cut into pieces of 20 mm in diameter and cleaned in a detergent solution (1.0 mL in 50 mL deionized water) under ultrasonic conditions for 30 minutes. The nets were further washed with deionized water in an ultrasonic bath and dried at 85 ºC.

1.2. Syntheses of $AlPO_4$-18 seeds and membranes

Nanosized $AlPO_4$-18 crystals were prepared in a synthesis solution with the molar ratio of $1.0Al_2O_3$:$3.16P_2O_5$:$3.16(TEA)_2O$:$186H_2O$ according to the literature.[36] The protocol was detailed as followed: first, Al(i-$C_3H_7O)_3$ was dissolved in an aqueous TEAOH solution. A clear solution was obtained after stirring for about 20 minutes at room temperature. Second, H_3PO_4 was added into above solution under stirring in a dropwise manner, and this solution was kept under stirring for another 1 h. Third, the mixture solution was filtrated by a PTFE syringe with 450 nm pores in order to remove any suspended matter. The obtained clear precursor was transferred to the autoclave and heated in a microwave reactor (XT-9906, Xintuo Analytical Instruments Co., LTD, Shanghai, China) at 800 W and 25 minutes to reach 180 ºC and 2.0 MPa, and then held for 5.0 minutes. The suspension was purified by repeated centrifugations (20000 rpm and 5 minutes for each run), and then washed to neutral. The seed solution (~10 g L^{-1} in water) was deposited dropwise on pre-cleaned nets, and net substrates were subjected to additional drying. Seeded substrates were placed vertically in a mother solution with the molar composition of $1.0Al_2O_3$:P_2O_5:$(TEA)_2O$:$160H_2O$, which was prepared by mixing Al(i-$C_3H_7O)_3$, TEAOH and H_3PO_4 with deionized water under vigorously stirring for 6 h. Crystallization was conducted at 200 ºC in a static oven for 24 h. The membranes were taken out after cooling down, washed with deionized water in an ultrasonic bath, and dried at 85 ºC. The organic templates in $AlPO_4$-18 membranes were removed by calcination in air at 550 ºC (heating ramp of 1.0 ºC min^{-1}) for 6 h.

1.3. Characterizations

The crystalline structures of $AlPO_4$-18 seeds and membranes were determined by X-ray diffractions on Rigaku SmartLab X-ray diffractometer with Cu-Kα radiation (λ = 1.5418 Å) running at a voltage of 40 kV and a current of 30 mA. The seeded and membrane layers were visualized on field emission scanning electron microscope equipped with energy-dispersive X-ray spectroscopy (FE-SEM, SU-8010, Hitachi). N_2 physical sorption was carried out at 77 K on Autosorb-iQ2 adsorptometer (Quantachrome Instruments). Water adsorption isotherms were recorded at 298 K on

the same type of apparatus (Autosorb-iQ2 adsorptometer). Prior to both measurements, $AlPO_4$-18 sample was degassed at 200 ºC for 12 h under vacuum.

1.4. Simulations

To determine water adsorption and diffusion behaviors in $AlPO_4$-18 at 298 K, the grand canonical Monte Carlo (GCMC) and molecular dynamics (MD) were performed respectively using the RASAP code developed in previous reports.[38-39]

In the simulations, the structure (2×2×2 unit cell) was treated as a rigid framework. The number of unit cells for $AlPO_4$-18 was chosen in order to meet the requirement that all of the perpendicular cell lengths are larger than twice the cutoff distance, which was set to 12 Å in our simulations. Guest water molecules were described by the TiP4P/Ew model,[40] a TiP4P model parameterized for Ewald sums. Lennard-Jones and Coulombic potentials were used to describe the interactions between host and guest. The like-pair parameters of atoms in $AlPO_4$-18 were taken from the literature (Table S2), and the unlike-pair parameters were determined using Lorentz-Berthelot combining rules.[41] The atomic point charges of $AlPO_4$-18 unit cell were calculated by using quantum mechanics (QM) method in DMol3 8.0[42] and the ones of water molecules were set as the description in the TiP4P/Ew model. Ewald method with precision of 10^{-6} was used to compute the charge interactions between atoms.

In GCMC simulations, each point of the isotherms at 298 K was obtained after equilibration runs of 50000 cycles followed by production runs of 300000 cycles. The MD simulations were performed in the NVT ensemble using the Nose-Hoover thermostats. A set of classical trajectories by integrating the equations of motion for a group of molecules was created through the MD method. The MD simulations were run for 4 ns with 0.5 ns equilibrium. A time step of 0.5 fs was used to integrate the equations of motion. In the equilibrium MD simulation, the self-diffusion coefficient D_s was evaluated by taking the slope of the mean-squared displacement (MSD)

Equation (1)

$$D_s = \frac{1}{2dN}\lim_{t\to\infty}\frac{d}{dt}\left\langle \sum_{i=1}^{N}\left(r_i(t) - r_i(0)\right)^2 \right\rangle$$

where N is the number of molecules, d is the spatial dimension of system, t is the simulation time and r_i is the center-of-mass of molecule i.

In order to investigate the transport properties of water molecules in $AlPO_4$-18, three water loadings (1, 8 and 396 water molecules per super cell marked respectively as m1, m8 and m396) were simulated through MD method. Here, m1 had one water molecule within the whole framework, m8 had eight water molecules within one large pore and the number of water molecules for m396 was chosen according to the GCMC simulation result under 2600 Pa.

In Dmol3 calculation process, exchange-correlation functions were calculated within a General Gradient Approximate (GGA) with the Perdew-Burke-Ernzerhof (PBE) functional.[46] The effective core potential (ECP) was adopted together with the double numerical (DNP) basis set. The Brillouin zone was sampled using a 3×3×2 Monkhorst-Pack k-point grid. The convergence criterion of the geometric optimization was set to be 1.0×10^{-5} Hartree for energy change, 2.0×10^{-3} Hartree/Å for the gradient, 5.0×10^{-3} Å for the displacement, and 1.0×10^{-6} eV for self-consistent field energy, respectively. Hirshfeld charge population analysis was adopted to obtain the charge distribution.[47]

1.5. Desalination tests

Prior to desalination, the membrane continuity was checked by single-gas permeations of H_2 and N_2 in a standard mode. Typically, single gas of H_2 or N_2 (each at 50 ml min^{-1}) was fed into the membrane chamber, the permeance of corresponding permeate gas was measured by an online gas chromatography (SCION 456GC). The H_2/N_2 selectivity was determined by the ratio of H_2 and N_2 pressures in the permeate over the corresponding ratio in the feed. The separation performances of $AlPO_4$-18 membranes for seawater desalinations were assessed using a batch pervaporation system, the apparatus of which was described in our previous report.[18] The supported $AlPO_4$-18 membrane was fixed in the membrane chamber and sealed with xylene resistant fluorocarbon O-rings, and connected to a vacuum line at the permeate side. The pressure on the feed side was kept at one atmosphere (~101 kPa). Single- (0.5M NaCl, 0.5M KCl, 0.5M $MgCl_2$, 0.5M $CaCl_2$) and multi-component (a mixture

containing 0.5M NaCl, 0.01M KCl, 0.05M $MgCl_2$, 0.01M $CaCl_2$) salt solutions were stored in a mixing tank and circulated using a small pump in order to minimize the concentration gradient. The experiments were performed at 22 ºC (25 ºC), 35 ºC, 45 ºC, 55 ºC, 65 ºC, the temperature of which was monitored by a thermocouple placed in the center of the membrane module. The permeated solution was collected in a liquid-nitrogen cooled trap after a preset time interval of ~7 h. The amount of permeated solution was calculated by the weight difference of the trap before and after each test. The concentrations of the feed and permeated solutions were analyzed by inductively coupled plasma atomic emission spectrometer (ICPAES, a Leeman-labs Prodigy High Dispersion ICP instrument). The separation characteristics were defined using the parameters of water flux (F, kg m^{-2} h^{-1}) and ion rejection (R, %) in the following:

Equation (2)

$$F = \frac{M}{S \times t}$$

Equation (3)

$$R = \frac{C_{i,feed} - C_{i,permeate}}{C_{i,feed}} \times 100\%$$

where M is the permeate mass (kg), S is the membrane area (m^2), t is the permeation time (h), i is the ion type, C_{feed} and $C_{permeate}$ are ion concentrations in the feed and permeated solutions (ppm), respectively. After each test, the membrane was easily regenerated by washing surface residues in water in an ultrasonic bath for 5.0 minutes.

2. Supporting figures and tables

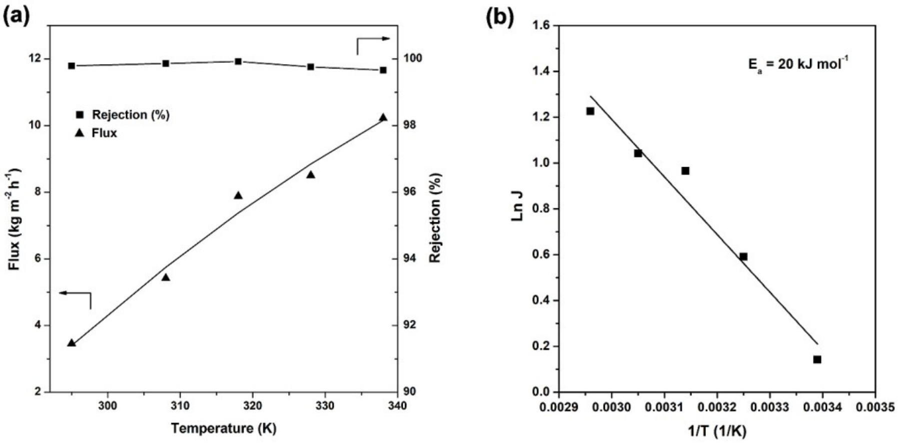

Fig. S1 (a) Water flux and Na^+ rejection degree in function of temperature (0.5 M NaCl, a pressure drop of ~1.0 atm) on the $AlPO_4$-18 membrane, and (b) calculated activation energy for water permeation based on the data in (a).

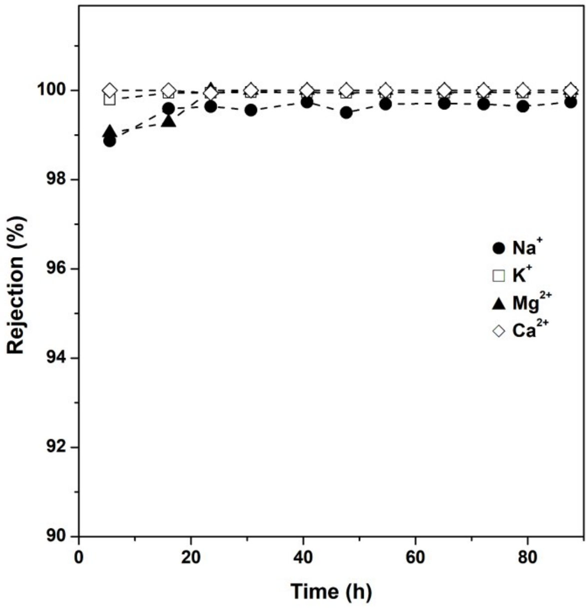

Fig. S2 The variations of rejection degrees for Na^+, K^+, Mg^{2+}, and Ca^{2+} cations (a simulated seawater solution containing 3.5% salts) in function of test time on the $AlPO_4$-18 membrane (test conditions: 298 K, feed pressure of 1.0 atm).

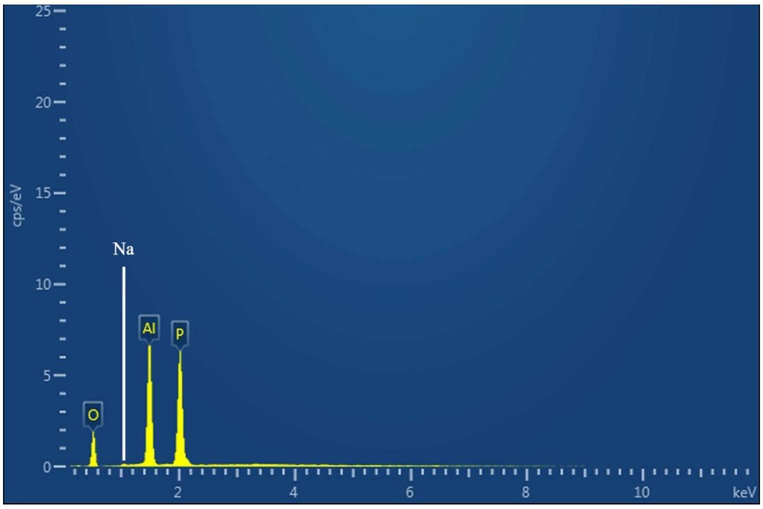

Fig. S3 Elemental mapping of the $AlPO_4$-18 membrane after desalination test measured by EDS.

Table S1 Textural data of chemical composition and pore structure for $AlPO_4$-18 collected from the membrane.

Sample	Al/P Ratio[a]	Surface Area (m^2 g^{-1})[b]	V_{total} (cm^3 g^{-1})[c]	V_{micro} (cm^3 g^{-1})[d]	Pore Width (Å)[e]
$AlPO_4$-18	1.02	754	0.647	0.283	8.23

[a] Aluminum-to-phosphorus ratio measured by ICP; [b] Brunauer-Emmett-Teller (BET) surface area estimated at $P/P_0 = 0.99$; [c] total pore volume calculated at $P/P_0 = 0.99$; [d] micropore volume determined by *t*-plot method; [e] pore width in diameter determined by NL-DFT method.

Table S2 Interaction potential parameters used for $AlPO_4$-18.

	σ (Å)	ε (K)
Al[a]	4.008	254.151
P[a]	3.695	153.497
O[b]	3.000	93.53

[a] C. A. Koh, T. Montanari, R. I. Nooney, S. F. Tahir and R. E. Westacott, *Langmuir*, 1999, **15**, 6043-6049; [b] M. Trzpit, M. Soulard, J. Patarin, N. Desbiens, F. Cailliez, A. Boutin, I. Demachy and A. H. Fuchs, *Langmuir*, 2007, **23**, 10131-10139.
